# Electroretinography Reveals Difference in Cone Function between Syndromic and Nonsyndromic *USH2A* Patients

**DOI:** 10.1038/s41598-017-11679-y

**Published:** 2017-09-11

**Authors:** Jesse D. Sengillo, Thiago Cabral, Kaspar Schuerch, Jimmy Duong, Winston Lee, Katherine Boudreault, Yu Xu, Sally Justus, Janet R. Sparrow, Vinit B. Mahajan, Stephen H. Tsang

**Affiliations:** 1 Jonas Children’s Vision Care, and Bernard & Shirlee Brown Glaucoma Laboratory, Department of Ophthalmology, Columbia University Medical Center, New York, NY USA; 2 Edward S. Harkness Eye Institute, New York-Presbyterian Hospital, New York, NY USA; 3 State University of New York Downstate Medical Center, Brooklyn, NY USA; 4 Department of Ophthalmology, Federal University of Espírito Santo, Vitoria, Brazil; 5 Department of Ophthalmology, Federal University of São Paulo, Sao Paulo, Brazil; 6 Department of Biostatistics, Columbia University, New York, NY USA; 7 Department of Ophthalmology, University of Montreal, Montreal, Canada; 8 Department of Ophthalmology, Xin Hua Hospital affiliate of Shanghai Jiao Tong University School of Medicine, Shanghai, China; 9 Department of Pathology & Cell Biology, Stem Cell Initiative (CSCI), Institute of Human Nutrition, College of Physicians and Surgeons, Columbia University, New York, NY USA; 10 Omics Laboratory, Byers Eye Institute, Department of Ophthalmology, Stanford University, Palo Alto, CA USA

## Abstract

Usher syndrome is an inherited and irreversible disease that manifests as retinitis pigmentosa (RP) and bilateral neurosensory hearing loss. Mutations in *Usherin 2A (USH2A)* are not only a frequent cause of Usher syndrome, but also nonsyndromic RP. Although gene- and cell-based therapies are on the horizon for RP and Usher syndrome, studies characterizing natural disease are lacking. In this retrospective analysis, retinal function of *USH2A* patients was quantified with electroretinography. Both groups had markedly reduced rod and cone responses, but nonsyndromic *USH2A* patients had 30 Hz-flicker electroretinogram amplitudes that were significantly higher than syndromic patients, suggesting superior residual cone function. There was a tendency for Usher syndrome patients to have a higher distribution of severe mutations, and alleles in this group had a higher odds of containing nonsense or frame-shift mutations. These data suggest that the previously reported severe visual phenotype seen in syndromic *USH2A* patients could relate to a greater extent of cone dysfunction. Additionally, a genetic threshold may exist where mutation burden relates to visual phenotype and the presence of hearing deficits. The auditory phenotype and allelic hierarchy observed among patients should be considered in prospective studies of disease progression and during enrollment for future clinical trials.

## Introduction

Usher syndrome is an inherited autosomal recessive condition affecting both hearing and vision, specifically manifesting as retinitis pigmentosa (RP) and bilateral neurosensory hearing loss^1–4^. It is the most common cause of deaf-blindness and currently irreversible. The retinal phenotype is characterized by night-blindness, a constricting visual field, and classic pigmentary degeneration seen on imaging^2, 5, 6^. Usher syndrome is categorized *clinically* into three types (Usher syndrome types 1–3) based predominantly on the onset and progression of hearing loss. Usher syndrome type 1 typically exhibits congenital hearing loss, childhood-onset RP, and possible vestibular dysfunction. Usher syndrome type 2 is characterized by mild, non-progressive hearing loss and early adulthood-onset of RP. Usher syndrome type 3 is the rarest, typically manifesting with progressive hearing loss, early-onset RP, and sporadic vestibular dysfunction^7, 8^. In addition to this traditional classification, the diagnosis can be refined with genetic testing, as mutations in at least 13 genes are known to cause Usher syndrome^9^. These genes are typically involved in ciliary transport or scaffolding proteins important for cellular structure and morphology^5, 10–15^.

Previously, Usher syndrome was thought to affect approximately 1 in 25,000 individuals, but recent epidemiologic data suggest a higher prevalence^16^. This could be due in part to cases in which there is a subtle hearing phenotype, resulting in an incomplete diagnosis of isolated RP. Interestingly, mutations in some genes leading to Usher syndrome can also manifest as nonsyndromic RP (NSRP) in other individuals, with mutations in *Usherin 2A* (*USH2A*) being a frequent cause of both^17–23^. Recently, a multi-center analysis of a large *USH2A* patient cohort found different visual outcomes in patients with Usher syndrome compared to NSRP^24^. Syndromic patients tended to exhibit worse visual outcomes as measured by subjective tests of visual function and harbored a higher proportion of truncating mutations. Another study also assessed syndromic and nonsyndromic *USH2A* patients but found no difference in retinal disease progression when comparing patients with the p.(Cys759Phe) amino acid substitution in USH2A, which is associated with NSRP, to patients with a p.(Glu767Serfs*21) frameshift in USH2A, which is implicated in Usher syndrome^6^. Differences in statistical modeling of progression, follow-up time, and the genotypes of interest in each study may account for the differing results, highlighting the need for further investigation. Assessing functional outcomes in syndromic and nonsyndromic *USH2A* patients is essential for patient management and counseling. Furthermore, understanding natural disease is necessary for future clinical trials so physicians can conclude if treatments are efficacious and which patients are most likely to benefit^7, 25^.

The present study sought to elucidate differences in cone function between Usher syndrome and NSRP due to mutations in the same *USH2A* gene. Patients were examined in a single electrophysiology clinic using objective visual function data, specifically the 30 Hz-flicker full-field electroretinogram (ffERG). The 30 Hz-flicker ffERG is a commonly used outcome measure of visual function and prognosis in RP patients^26–31^. This particular ffERG measures cone function by exposing the patient to a light stimulus at a rate of 30 flashes per second after the retina is bleached to suppress rod responses, resulting in a predominantly L- and M-cone response. While the rod response aids in diagnosis and is typically severely diminished or completely extinguished in Usher syndrome and RP, the 30 Hz-flicker is useful for assessing retinal function in clinical practice, as cones are affected later in the disease course^26, 27, 31, 32^. The mutation spectrum was also assessed in this cohort of *USH2A* patients and compared to the findings of previous studies.

## Results

### Clinical data

**Figure 1:**
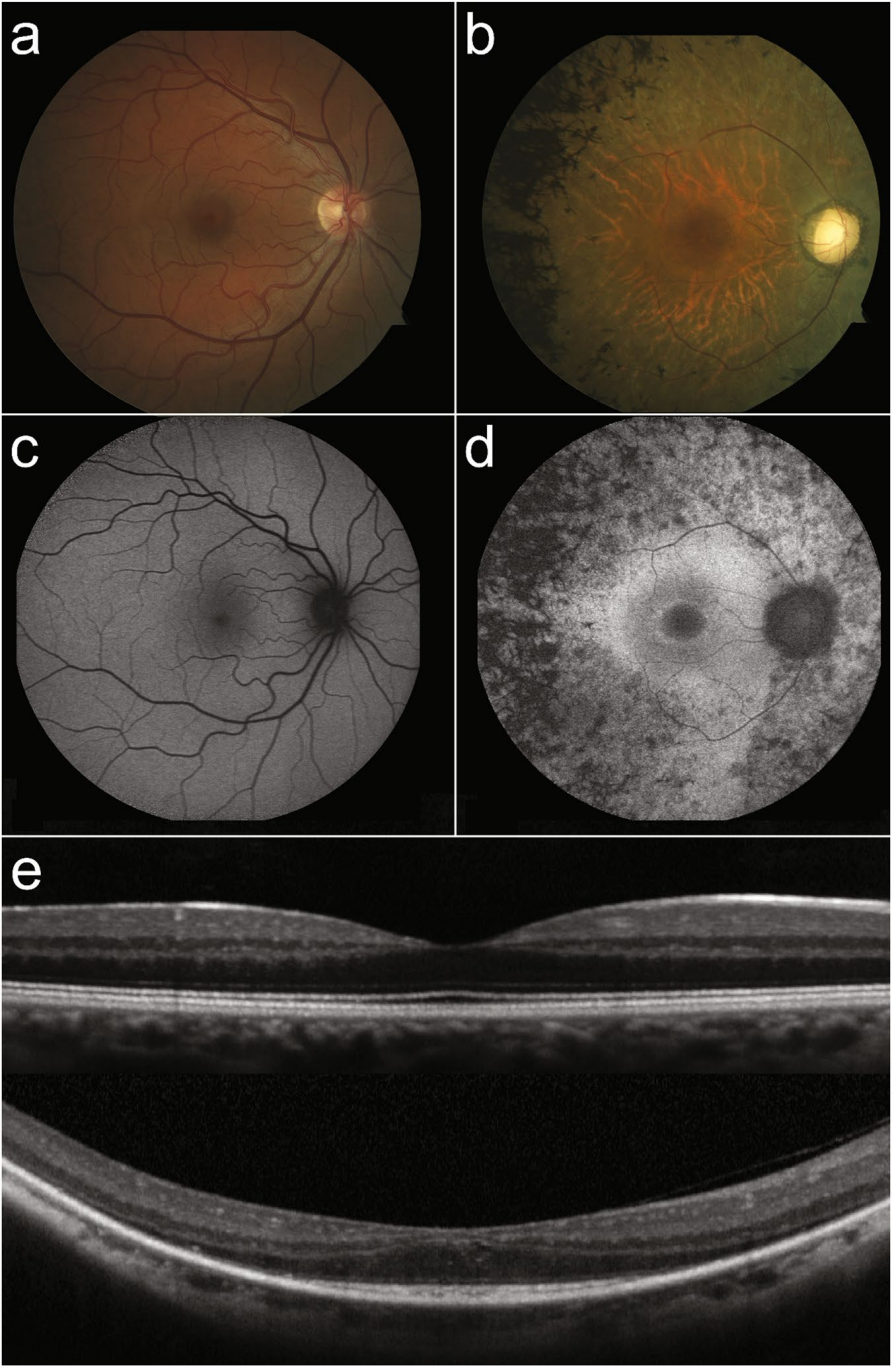
Retinal imaging of a patient with NSRP due to mutations in USH2A compared to a healthy individual. Color fundus photo of the posterior pole of a healthy individual (**a**) compared to a patient (P18) with NSRP (**b**), showing extensive intraretinal pigment migration observed temporally. SW-AF imaging reveals a characteristic foveal autofluorescent ring in P18 (**d**), which is absent in the healthy individual (**c**). SD-OCT of a healthy individual shows intact retinal structure (**e**, *top*) compared to P18 (**e**, *bottom*). Note the thinning of outer retinal layers in the periphery and the sparing of the ellipsoid zone in the central macula in P18.

A total of 10 patients were diagnosed with Usher syndrome type IIA and 10 patients were diagnosed with recessive NSRP based on clinical history, family history, dilated fundus exam, presence or absence of subjective hearing loss, and genetic confirmation of two or more *USH2A* mutations. The diagnosis was supported by multimodal retinal imaging (Fig. 1) and/or ffERG scotopic b-wave responses that were decreased to a greater extent than photopic responses, indicative of a rod-cone dystrophy. Clinical and genetic findings of the cohort are provided in Tables 1 and 2. The average age of the Usher syndrome group was 33 (n = 10) compared to 44 (n = 10) in the NSRP group (p = 0.16). Four eyes had best-corrected visual acuity (BCVA) of 20/100 or worse in Usher syndrome patients. In NSRP patients, one eye was less than 20/100, which was in the context of cystoid macular edema (CME). Three of ten Usher syndrome patients had a bulls-eye pattern of macular atrophy or significant maculopathy in both eyes. One NSRP patient had a bulls-eye pattern of atrophy bilaterally. On fundoscopy, nine Usher syndrome patients had evident intraretinal pigment migration. In the youngest patient (P10), no intraretinal pigment migration was observed. In the NSRP group, seven patients had intraretinal pigment migration. No patient showed evidence of CME on SD-OCT in either eye of Usher syndrome patients, compared to three NSRP patients. Two patients (P8 and P14) underwent previous audiometry for which P8 (Usher syndrome) was found to have bilateral neurosensory hearing loss. P14 (NSRP) underwent audiometry to screen for hearing loss after receiving results of genetic testing, which was in the normal range.

**Table 1:** Clinical Characteristics of *USH2A* Patients.

	ID	Gender	Age	BCVA (OD)	BCVA (OS)	Pigment	CME (OD, OS)	Comments
Syndromic	P1	M	25	20/20	20/20	+	−	
	P2	M	17	20/20	20/20	+	−	
	P3	M	45	20/20	20/50	+	−	
	P4	F	24	20/20	20/400	+	−	Macular Hole OS
	P5	F	28	20/20	20/20	+	−	
	P6	F	24	20/30	20/50	+	−	
	P7	M	59	20/125	20/LP	+	−	Bulls-eye OU
	P8	F	36	20/25	20/40	+	−	Bulls-eye OU
	P9	M	57	20/100	20/80	+	−	Maculopathy
	P10	M	19	20/60	20/40	−	−	
Nonsyndromic	P11	F	30	20/20	20/20	+	−	
	P12	M	16	20/20	20/20	−	−	
	P13	M	68	20/40	20/60	−	−	Bulls-eye OU
	P14	F	31	20/20	20/20	+	−	
	P15	F	25	20/30	20/25	+	+, +	
	P16	M	56	20/50	20/25	+	−	
	P17	F	57	20/20	20/20	+	−	
	P18	F	52	20/30	20/25	+	+, −	
	P19	M	50	20/25	20/20	−	−	
	P20	M	55	20/200	20/80	+	+, −	

BCVA, best-corrected visual acuity; OD, right eye; OS, left eye; OU, both eyes; CME, cystoid macular edema.

**Table 2:** Genetic Profile of *USH2A* Patients.

	ID	Gender	Age	*USH2A* mutation	Protein Change	Segregation
Syndromic	P1	M	25	c.1724G > A	p.(Cys575Tyr)	—
				c.2299delG	p.(Glu767Serfs*21)	
	P2	M	17	c.3713C > G	p.(Thr1238Arg)	—
				c.9459C > A	p.(Cys3153*)	
	P3	M	45	c.8442_8443insT	p.(Thr2815Tyrfs*20)	—
				c.1000C > T	p.(Arg334Trp)	
	P4	F	24	c.11918delC	p.(Ala3973Valfs*11)	*In trans*
				c.10712C > T	p.(Thr3571Met)	
	P5	F	28	c.2299delG	p.(Glu767Serfs*21)	*Homozygous*
				c.2299delG	p.(Glu767Serfs*21)	
	P6	F	24	c.13112_13115delAAAT	p.(Gln4371Argfs*19)	*In trans*
				c.13943delG	p.(Gly4648Aspfs*30)	
	P7	M	59	c10712C > T	p.(Thr3571Met)	*Homo*
				c10712C > T	p.(Thr3571Met)	
	P8	F	36	c10712C > T	p.(Thr3571Met)	*In trans*
				c.4711G > C	p.(Ala1571Pro)	
	P9	M	57	c.2299delG	p.(Glu767Serfs*21)	—
				c.12574C > T	p.(Arg4192Cys)	
	P10	M	19	c.15520-22_15524del27	p.(?)	—
				c.6049 + 2T > G	p.(?)	
Nonsyndromic	P11	F	30	c.9676C > T	p.(Arg3226*)	—
				c.1478A > G	p.(Tyr493Cys)	
	P12	M	16	c.4251 + 1G > A^ǂ^	p.(?)	*ǂIn trans*
				c.13223T > C	p.(Val4408Ala)	
				c.13231C > G	p.(Leu4411Val)	
	P13	M	68	c.2276G > T	p.(Cys759Phe)	*Homozygous*
				c.2276G > T	p.(Cys759Phe)	
	P14	F	31	c.10073 G > A	p.(Cys3358Tyr)	—
				c.10759C > T	p.(Gln3587*)	
	P15	F	25	c.13010C > T	p.(Thr4337Met)	—
				c.9740-1G > T	p.(?)	
	P16	M	55	c.10073G > A	p.(Cys3358Tyr)	*Homozygous*
				c.10073G > A	p.(Cys3358Tyr)	
	P17	F	57	c.14287G > A	p.(Tyr493Cys)	*In trans*
				c.3476C > T	p.(Pro1159Leu)	
	P18	F	52	c.895delC	p.(Gln299Asnfs*37)	—
				c.5848A > G	p.(Thr1950Ala)	
	P19	M	50	c.13491_13499dupTACTCTCAC	p.(Thr4498_Thr4500dup)	*Homozygous*
				c.13491_13499dupTACTCTCAC	p.(Thr4498_Thr4500dup)	
	P20	M	55	c.12575G > A	p.(Arg4192His)	*In trans*
				c.8682-9A > G	p.(?)	

### Electrophysiology

**Figure 2:**
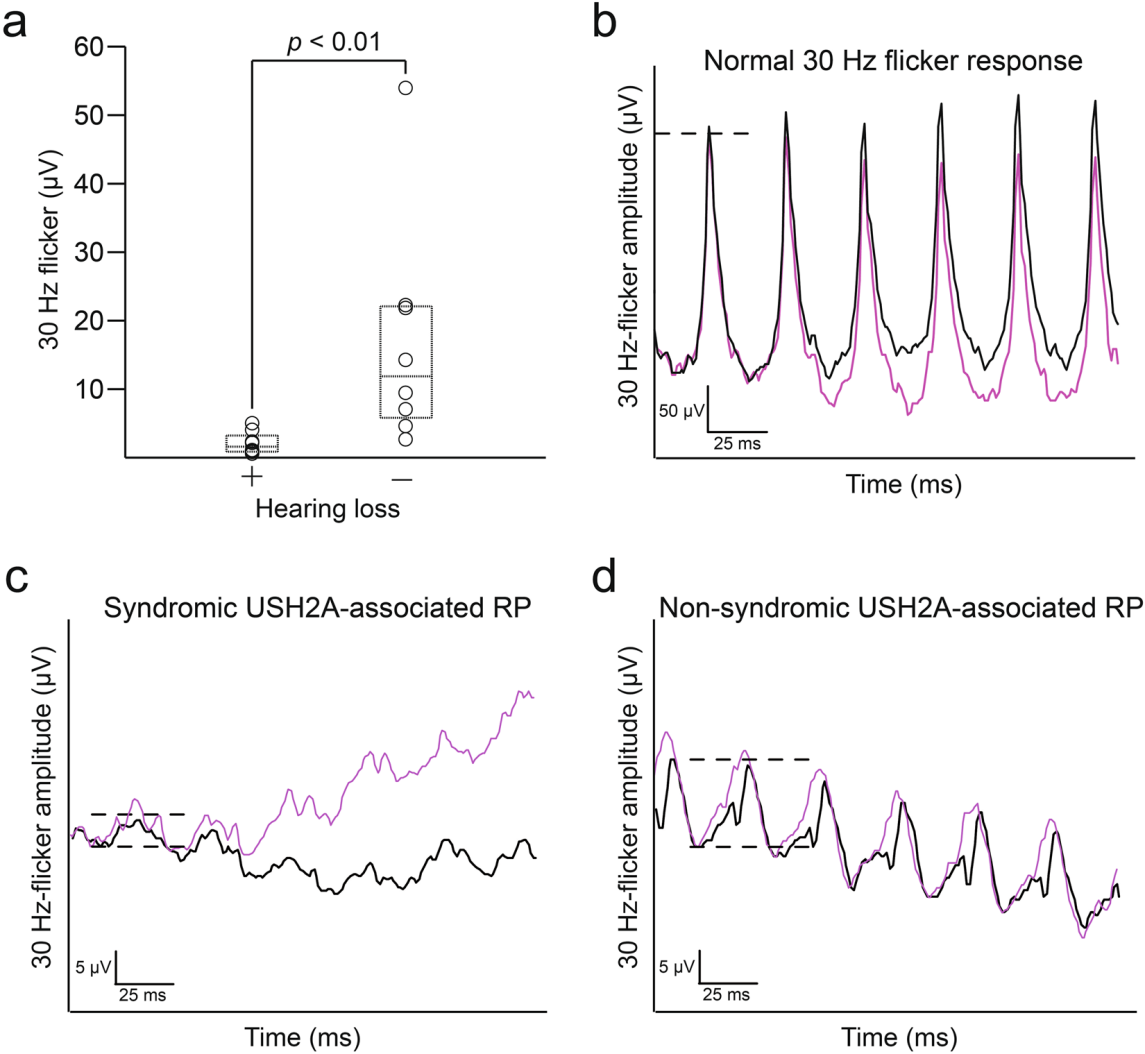
Differences in 30 Hz flicker electroretinogram between syndromic and nonsyndromic USH2A patients. (**a**) Modified box-and-whisker plots with individual patient data points reveal a statistically significant difference in 30 Hz-flicker amplitudes between Usher syndrome and NSRP patients. Representative 30 Hz-flicker ERGs for a normal patient (**b**), Usher syndrome patient (P5, **c**), and NSRP patient (P11, **d**). P5 had the highest average 30 Hz-flicker amplitude among Usher syndrome patients; P11 had a 30 Hz-flicker amplitude average nearest to the median of the NSRP group. Circles, individual patient data points; black lines, right eye; purple lines, left eye; dashed lines in (**b**–**d**), illustrate amplitude difference between peak and trough.

Results of ffERG testing are presented in Table 3. Eight patients in each group underwent electroretinography. NSRP patients had mean 30 Hz-flicker amplitudes of 17.04 ± 16.7 μV (mean ± SD), compared to Usher syndrome patients, 2.13 ± 1.64 μV (Fig. 2). 30 Hz-flicker amplitudes were compared between groups using the Wilcoxon rank-sum test (*p* = 0.0023). Seven of eight patients in the Usher syndrome group required Burien-Allen (BA) contact lens electrodes compared to three of eight NSRP patients. All others used Dawson, Trick, and Litzkow (DTL) recording electrodes. Of the 10 total patients whose recordings were performed with BA contact lens electrodes, four (P3, P4, P6, P8) also had prior DTL recordings performed during the same visit; all four DTL recordings were nondetectable. Five of eight Usher syndrome patients had an implicit time delay in 30 Hz-flicker recordings compared to all NSRP patients (p = 0.20). Scotopic b-wave amplitudes were recorded in a total of 12 patients (24 eyes). Scotopic b-waves were extinguished in all 10 eyes of Usher syndrome patients and 10 of 14 eyes of NSRP patients.

**Table 3:** Electroretinography of *USH2A* Patients.

	*ID*	Scotopic B-wave Amplitude (µv)		30 Hz Flicker Amplitudes (µv)		30 Hz Flicker Implicit Time (ms)		*Electrode*
		*OD*	*OS*	*OD*	*OS*	*OD*	*OS*	
Syndromic	P1	—	—	2.4	2.3	19	21	*ba*
	P2	*ext*	*ext*	1.0	1.2	38^d^	33^d^	*ba*
	P3	*ext*	*ext*	1.7	2.7	28^d^	31^d^	*ba*
	P4	*ext*	*ext*	1.7	6.5	34^d^	33^d^	*ba*
	P5	*ext*	*ext*	2.8	7.2	28	28	*dtl*
	P6	*ext*	*ext*	0.7	0.4	33^d^	32^d^	*ba*
	P7	—	—	1.3	0.7	28	27	*ba*
	P8	—	—	0.8	0.8	27^d^	28^d^	*ba*
	P9	—	—	—	—	—	—	—
	P10	—	—	—	—	—	—	—
Nonsyndromic	P11	*ext*	*ext*	12.9	15.7	40^d^	39^d^	*dtl*
	P12	*ext*	*ext*	5.0	4.2	44^d^	45^d^	*dtl*
	P13	20.3	26.8	7.3	11.6	40^d^	41^d^	*dtl*
	P14	*ext*	*ext*	22.6	22.0	42^d^	43^d^	*dtl*
	P15	—	—	19.2	24.6	30^d^	30^d^	*ba*
	P16	109.1	141.0	43.5	64.6	31^d^	30^d^	*dtl*
	P17	*ext*	*ext*	5.1	9.0	33^d^	33^d^	*ba*
	P18	*ext*	*ext*	3.0	2.4	27	31^d^	*ba*
	P19	—	—	—	—	—	—	—
	P20	—	—	—	—	–	—	—

OD, right eye; OS, left eye; *ext*, extinguished; −, not performed; *ba*, Burian-Allen contact lens electrode; *dtl*, DTL recording electrode; ^d^delayed.

### Genetic analyses

The genetic profile of all patients are provided in Table 2, and each *USH2A* mutation identified in the cohort is tabulated in Supplemental Table 1. All patients had family histories consistent with an autosomal recessive inheritance pattern. No patients in this cohort were related to each other. P19 reported a family history of parental consanguinity. The distribution of severe mutations was assessed between groups. A severe mutation was defined as a nonsense mutation or an indel (insertion or deletion) resulting in an out-of-frameshift. The percentage of Usher syndrome patients having at least one severe mutation was 70% compared to 30% of NSRP patients (*p* = 0.089). In the Usher syndrome group, 45% (9/20) of alleles had a nonsense mutation or an indel resulting in an out-of-frameshift compared to 15% (3/20) of NSRP patients. The odds ratio for an allele containing a frameshift or nonsense mutation in the Usher syndrome group compared to the NSRP group was 4.6 (confidence interval: 1.1 to 20, *p* = 0.039). Two patients in the Usher syndrome group harbored two truncating alleles compared to no patients in the NSRP group. Mutations were known to be *in trans* or homozygous in 5 of 10 Usher syndrome patients and 6 of 10 NSRP patients. The phase of the two *USH2A* mutations was unknown in the remaining patients. A 27 base-pair deletion was identified in P10 (Usher syndrome), which included the loss of a splice-site acceptor. As it is not clear if this allele is affected more by aberrant splicing or a frameshift, it was not counted as severe to maintain a conservative analysis. A homozygous, in-frame duplication was identified in P19 (NSRP) and thus not considered severe based on our criteria, as the amino acid reading frame is unaffected. One of 10 patients in the Usher syndrome group and three of 10 patients in the NSRP group harbored at least one variant affecting splicing. One patient (P12) was identified to have three variants in *USH2A*, in which the variant affecting splicing was found to be *in trans* to the two missense variants.

### Structural measurements

**Figure 3:**
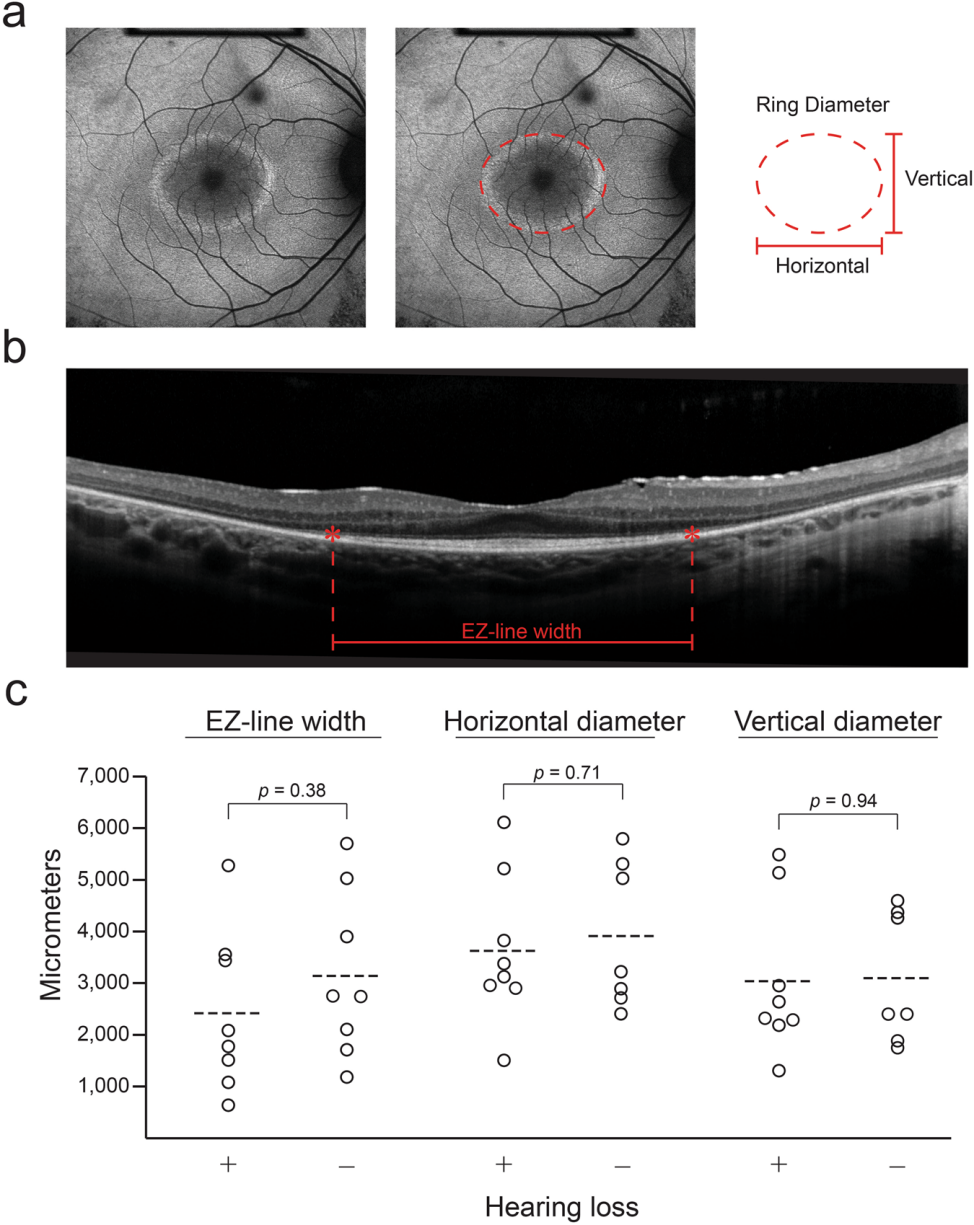
Comparison of structural measurements on retinal imaging between syndromic and nonsyndromic USH2A patients. Mean horizontal and vertical autofluorescent ring diameters measured on SW-AF (**a**) and ellipsoid zone line length measured on SD-OCT (**b**) were compared between syndromic and non-syndromic *USH2A* patients (**c**). Circles, individual patient data points; dashed lines, group mean.

Short wavelength autofluorescent (SW-AF) and spectral-domain optical coherence tomography (SD-OCT) imaging were obtained for all patients. Four patients (P7, P9, P13, P20) had bilateral advanced disease, rendering the ellipsoid zone (EZ) line length and AF ring diameters unmeasurable; the left eye of one patient (P4) had a macular hole that prevented accurate EZ-line and ring visualization. One patient (P17) had a granular AF ring that was not quantifiable. CME was observed on SD-OCT for three patients (P15, P18, P20) but did obscure the EZ-line. No patients warranted exclusion due to poor imaging quality. For all eyes with an AF ring, it was visible within the central 30 degrees of the retina. AF ring diameters were generally symmetrical between eyes, and 15 of 16 patients had horizontal diameters that were larger than vertical diameters. Mean EZ-line length between both eyes were 2425 µm for Usher syndrome patients and 3145 µm for NSRP patients (*p* = 0.38); Usher syndrome patients had mean horizontal and vertical ring diameters of 3633 μm and 3044 μm, respectively, compared to a horizontal diameter of 3915 μm (*p* = 0.71) and vertical diameter of 3101 μm (*p* = 0.94) in NSRP patients (Fig. 3).

### Case Study

A 41-year-old female with RP and two confirmed pathogenic *USH2A* mutations, c.10073G > A and c.920_923dupGCCA, did not have an auditory phenotype documented on previous intake forms or clinic notes. As subjective hearing loss could not be confirmed, this patient was excluded from analyses (see methods). Of interest, she did undergo previous audiometry for the work-up of tinnitus, which was found to be within the normal range for all tested frequencies. FfERG scotopic b-waves and 30 Hz-flicker amplitudes for this patient were higher than all patients in the cohort (Supplemental Table 2). Multimodal retinal imaging was performed in this patient, which was consistent with RP (Supplemental Fig. 2).

## Discussion

Usher syndrome presents with considerable genetic and phenotypic variability between individuals. Mutations in at least 13 genes are known to cause Usher syndrome but may manifest as a different clinical entity in other patients. In addition to *USH2A*, mutations in *CLRN1* are also known to cause both Usher syndrome and NSRP^33^. Furthermore, a longer list of genes are associated with both Usher syndrome and nonsyndromic recessive deafness (*CDH23*, *CIB2*, *DFNB31*, *MYO7A*, *PCDH15*, and *USH1C*)^5^. In these cases, differences in the mutational spectrum may relate to disease manifestation. For *MYO7A* and *CDH23*, truncating mutations are reported to be more commonly associated with Usher syndrome, while ‘leaky’ splice site and missense mutations manifest as nonsyndromic deafness^34, 35^. RP with hearing deficits have also been reported in the context of X-linked *RPGR* mutations, albeit less frequently^36^. The present study focused specifically on Usher syndrome and recessive NSRP due to mutations in *USH2A*, and is first to elucidate differences in cone function between these groups.

Overall, our data suggest that patients with Usher syndrome due to *USH2A* mutations have a more attenuated 30 Hz-flicker response compared to their NSRP counterparts (Fig. 2). NSRP patients in this retrospective analysis had a mean 30 Hz-flicker amplitude of 17 µV at an average age of 44 years old (n = 8). Usher syndrome patients in our cohort had inferior cone function with a mean 30 Hz-flicker amplitude of 2.1 μV at a younger average age of 33 years old (n = 8). 30 Hz-flicker ffERGs are a useful outcome measure to assess, as scotopic responses are affected earlier and often already extinguished in both RP and Usher syndrome at presentation. Photopic 30 Hz-flicker response is an outcome in previous landmark clinical trials assessing the efficacy of dietary supplements for RP^27–29^ and published actuarial tables provide conversions for 30 Hz-flicker amplitudes into prognoses for patient counseling^26^. Together, these data suggest that the presence of subjective hearing loss in *USH2A* patients may be predictive of worse cone function and thus visual phenotype. Interestingly, these large differences in retinal function did not corroborate structural measurements obtained from multimodal imaging in both groups, which would have been predicted based on the stark difference in 30 Hz-flicker amplitudes (Fig. 3). Future comparisons may require more subjects to detect a potential statistical difference. Alternatively, the difference in ERG amplitudes may reflect the number of healthy cone photoreceptors in the peripheral retina, as an ERG represents an average of the entire fundus. In contrast, structural measurements in this study were within the macula, which is affected later in the disease course. Lastly, functional loss precedes structural changes, hence an ERG is a more sensitive test to detect changes in the retina. As a result, structural measurements for both groups may be assessed more effectively as a progression rate over a longitudinal follow-up, as opposed to in cross-section.

The Usher syndrome group was more likely to be enriched with disease alleles containing either an indel or nonsense mutation, which generally has a more deleterious effect on protein structure (Table 1). Allelic hierarchy has been reported previously and was confirmed in this study^19^. It is possible that a genetic threshold exists in which the combined burden of the two alleles is severe enough to cause hearing loss in addition to the retinal phenotype. Alternatively, differential disruption of USH2A isoforms or their expression patterns in photoreceptor and inner ear cells may account for variable hearing deficits. The genetic heterogeneity and frequent occurrence of compound heterozygous mutations in *USH2A* patients, however, make it difficult to confirm this hypothesis.

In our cohort of 20 patients, exactly 31 unique disease-causing variants were identified in *USH2A*. Of these, the most common variants were c.2299delG, c.10073G > A, and c.10712C > T. The c.2299delG variant is the most frequent disease-causing variant of Usher syndrome in multiple studies^37–39^, and was not found in the NSRP group. c.10712C > T mutations were only identified in Usher syndrome patients in this cohort, and previous studies have also associated this particular variant with RP and an auditory phenotype^19^. This missense mutation causes the reading frame to be interrupted by a premature STOP codon; mRNA produced might be targeted for nonsense-mediated decay. Interestingly, Lenassi *et al*. found the c.10073G > A, c.2276G > T, and c.12575G > A mutations to be ‘retinal disease specific’ and hypothesized the presence of one of these mutations leads to a nonsyndromic manifestation, largely independent of the second mutation^19^. Our mutational analysis found this to be possible. P13 and P16, both NSRP, harbored homozygous c.10073G > A and c.2276G > T variants, respectively. Similarly, the presented case study featured a patient with the ‘retina-specific’ c.10073G > A mutation^17, 19, 21^. This patient had a normal audiogram across all frequencies in addition to scotopic B-wave and 30 Hz-flicker amplitudes that were higher than all patients in the cohort.

There are previous studies with contrasting findings. Sandberg *et al*. compared patients with the p.(Cys759Phe) amino acid substitution in USH2A, which is known to cause NSRP, with patients that had a p.(Glu767fs*21) truncating mutation, known to cause Usher syndrome. No differences in disease progression were detected^6^. Our cohort observed only one patient (P13, NSRP) with the p.(Cys759Phe) substitution in USH2A and three patients (P1, P5, P9) with the p.(Glu767fs*21) truncating mutation, preventing a meaningful comparison within this study. However, conclusions from the present study are in accordance with the previously mentioned multi-center cohort study performed in Europe that found Usher syndrome patients to be diagnosed earlier, became visually impaired sooner by subjective functional testing, and have a higher frequency of truncating mutations compared to NSRP patients^24^. Visual prognoses were determined in their cohort with visual acuity and field measurements, which were superior in NSRP. Our retrospective analysis confirms a severe visual phenotype in Usher syndrome, elucidates differences in visual function with an *objective* test, and is the first to document differences in ffERG cone responses between these populations, an important index in RP research and clinical practice.

There are limitations to our study. Patients underwent ffERG testing with BA contact lens or DTL recording electrodes. Four Usher syndrome patients in this cohort underwent recordings with both DTL-recording electrodes and BA contact lenses in the same visit, with undetectable waveforms in the former. The increased use of BA contact lens electrodes in the Usher syndrome group likely decreased the potential difference between groups, which was still statistically significant. Prospective investigations and clinical trials would benefit from the use of a single type of recording electrode to compare cone function over time. Another limitation is the aforementioned genetic heterogeneity of Usher syndrome and RP, which lends smaller cohorts difficult to interpret. Of the 20 patients in this cohort, 4 were homozygous for *USH2A* mutations. In compound heterozygotes it is difficult to parse the contribution of a specific mutation to disease phenotype without a larger cohort. Lastly, using subjective hearing loss to diagnosis either Usher syndrome or NSRP likely leads to misclassification of patients with a subtle hearing phenotype that is undetectable to the patient. However, using subjective hearing loss as a criterion was useful in stratifying differences in auditory phenotype and can guide genetic screening and counseling. Still, a more sophisticated and quantifiable measure of hearing loss may reveal other correlations between particular mutations and auditory or visual manifestations. Future investigations should consider comparing visual outcomes to a quantifiable measure of auditory function, such as a standardized audiometry.

There is currently no cure for Usher syndrome. Standard management typically involves monitoring complications of RP and providing assistive hearing devices such as hearing aids or cochlear implants^40^. However, after previous demonstration of safety and efficacy in macaques, clinical trials for Usher syndrome type IB are underway that involve subretinal administration of UshStat® (EIAV-CMV-*MYO7A*) (NCT01505062)^41^. Recently, a preclinical anti-sense oligonucleotide has also shown promise in an Usher syndrome type 1C mouse model after being identified in an *in vitro* screen. By blocking an aberrant splice site, vestibular and auditory rescue is achieved in this model^42^. Specific to *USH2A*, investigators corrected the effects of a deep intronic mutation in patient-derived fibroblasts^43^. With gene- and cell-based therapies becoming a reality, studies characterizing natural disease become essential for assessing treatment efficacy. This study not only provides objective functional data comparing retinal function between Usher syndrome and NSRP caused by mutations in the same gene, but may also prove useful for retina specialists counseling *USH2A* patients.

## Methods

### Subjects

This retrospective analysis was approved by the Edward S. Harkness Eye Institute and Columbia University Internal Review Boards and adhered to the tenets of the Declaration of Helsinki. The data presented in this study, including images and genetic testing results, are not identifiable to individual patients. Informed consent for participation was obtained as outlined in the Columbia University Medical Center IRB-approved protocol, AAAR0284. Patients diagnosed with Usher syndrome or NSRP at the Harkness Eye Institute electrodiagnostics clinic were considered for inclusion in electroretinogram or mutational analyses based upon: (1) if they harbored at least two pathogenic or likely pathogenic *USH2A* variants and (2) had prior documentation of the presence or absence of subjective hearing loss on clinic notes or patient intake forms. It is important to note that all patients in the NSRP group reported no hearing loss on an intake form or during the clinic visit as per the physician note, meaning the auditory phenotype was not assumed to be normal if it was not stated explicitly. Twenty-seven patients presenting to clinic between 2010 and 2016 were found to have at least two *USH2A* mutations. Out of the 27 patients, seven were excluded from analyses for the following reasons: *USH2A* variants occurred *in cis* (one patient); two *USH2A* variants were reported as benign (one patient); other identified gene variants could reasonably account for disease phenotype with no accompanying co-segregation analysis (three patients); there was a confounding history of subjective hearing loss, which is the independent variable in each analysis performed (one patient); or there was no documentation of the presence or absence of hearing loss (one patient). The 20 included *USH2A* patients were analyzed in two groups, Usher syndrome (n = 10) and NSRP (n = 10). Multimodal imaging was acquired in all patients and a total of 16 patients underwent ffERG testing.

### Genetic analyses

For all patients, DNA was isolated from whole blood lymphocytes for sequencing. Most patients (15 of 20) had genetic testing for a panel of genes known to cause Usher syndrome or RP by next generation sequencing and subsequent confirmation with Sanger sequencing at the Casey Eye Institute Diagnostic Laboratory (Portland, Oregon), Molecular Vision Laboratory (Hillsboro, Oregon), or Columbia University Medical Center Pathology Department (New York, NY). Whole exome sequencing was performed on three patients by the Columbia University Medical Center Pathology Department (New York, NY). The remaining two patients had genetic testing performed at the National Institute on Deafness and Other Communication Disorders (Bethesda, Maryland) and the John and Marcia Carver Nonprofit Genetic Testing Laboratory (Iowa City, IA). *USH2A* variants were analyzed by Alamut software version 2.2 (Interactive Biosoftware, Rouen, France).

### Electroretinography

FfERGs were acquired with the Diagnosys Espion Electrophysiology System (Diagnosys LLC, Lowell, MA, USA) from both eyes using DTL recording electrodes in accordance to the International Society for Clinical Electrophysiology of Vision (ISCEV) standards in scotopic and photopic states^44, 45^. When the 30 Hz-flicker amplitudes were lower than five microvolts or predicted to be lower than five microvolts based on exam, Burian-Allen contact lens electrodes were used to record the 30 Hz-flicker responses that are subsequently processed through narrow band-passed filtering with computed averaging^26, 44, 46, 47^. The amplitudes obtained from both eyes were measured by a single user with Espion V6.0.56 software (Diagnosys LLC, Lowell, MA, USA), which calculates the amplitude from trough to peak of the first visible waveform, as performed previously^46, 48–50^. Amplitudes of both eyes were averaged and compared between the two groups.

### Imaging

Fundus SW-AF and SD-OCT images were obtained in all patients after dilation using Spectralis HRA + OCT device (Heidelberg Engineering, Heidelberg, Germany). SW-AF images were acquired using a 30-degree field of view with 1536 × 1536 pixel resolution. SW-AF imaging utilized a 521nm barrier filter and 486nm wavelength stimulus. The external horizontal and vertical ring diameter of SW-AF imaging was measured manually using Spectralis software (Heidelberg Eye explorer, software version 1.9.10, Heidelberg Engineering, Heidelberg, Germany). SD-OCT was obtained with an 870nm light source and automatic real-time registration of the fundus infrared reflectance image. Horizontal scans (high resolution mode, 9mm, ART, average of a minimum of 50 images) through the fovea provided a view of the EZ-line and was measured manually using Spectralis software. All measurements were obtained by a single observer (T.C), who was masked to whether the patient was in the Usher syndrome or NSRP group.

### Statistical analyses

All statistical analyses were performed using Stata/IC software version 13.0 (StataCorp LLC, College Station, Texas). 30 Hz-flicker ffERG amplitudes were compared between Usher syndrome and NSRP patients using a Wilcoxon-rank sum test. Right and left eyes were averaged prior to statistical analysis. To compare the distribution of patients harboring at least one frameshift (indel) or nonsense mutation, a Fisher’s exact test was used (Table 4, row 4). To account for a possible correlation of observations from the same subject, the odds of having either an indel or nonsense mutation in one allele was compared between both groups by fitting logistic regression models using generalized estimating equations. In this case, the outcome variable was whether an allele had either a nonsense mutation or an indel resulting in an out-of-frame shift (Table 4, row 5). Structural measurements acquired from SD-OCT (EZ-line length) and SW-AF imaging (horizontal and vertical ring diameters) were compared using a two-tailed Student’s *t* test with unequal variance. Measurements for the right and left eye were averaged prior to comparing groups for all indices. A *p* value less than 0.05 was considered statistically significant in all results reported. All values are expressed as mean ± standard deviation (SD) unless otherwise indicated.

**Table 4:** Summary Statistics for Mutation Analysis.

	Syndromic	Nonsyndromic	Odds ratio (CI)	P Value
N number	10	10		
Males, Females	6, 4	5, 5		
Age (mean ± SD)	33 ± 14	44 ± 16		*p* = 0.16
% of Patients with indels or nonsense mutations	70 (7/10)	30 (3/10)	6.4 (0.5, 128)	*p* = 0.09^†^
% of Alleles with indels or nonsense mutations	45 (9/20)	15 (3/20)	4.6 (1.1, 20)	*p* = 0.04^‡^

^†^Determined by Fisher’s exact test. ^‡^Determined by logistic regression models. Indel; insertion or deletion leading to an out-of-frame shift.

### Data Availability

All data generated or analyzed for this study are included in this published article.

## Electronic supplementary material

Supplementary Information
