# Supplementary material for: Electroretinography Reveals Difference in Cone Function between Syndromic and Nonsyndromic USH2A Patients
Source: Sci Rep. 2017 Sep 11;7:11170. doi: 10.1038/s41598-017-11679-y (PMC5593892; doi:10.1038/s41598-017-11679-y)
Supplement: Supplementary file 1 — Supplementary Information [file 41598_2017_11679_MOESM1_ESM.pdf]

Supplementary Information

Electroretinography Reveals Difference in Cone Function between Syndromic and Nonsyndromic *USH2A* Patients

Jesse D Sengillo[1,2,3], Thiago Cabral[1,2,4,5], Kaspar Schuerch[1,2], Jimmy Duong[6], Winston Lee[1,2], Katherine Boudreault[1,2,7], Yu Xu[8], Sally Justus[1,2], Janet R Sparrow[2,9], Vinit B Mahajan[10], Stephen H Tsang[1,2,9*]

[1]Jonas Children's Vision Care, and Bernard & Shirlee Brown Glaucoma Laboratory, Department of Ophthalmology, Columbia University Medical Center, New York, NY, USA
[2]Edward S. Harkness Eye Institute, New York-Presbyterian Hospital, New York, NY, USA
[3]State University of New York Downstate Medical Center, Brooklyn, NY, USA
[4]Department of Ophthalmology, Federal University of Espírito Santo, Vitória, Brazil
[5]Department of Ophthalmology, Federal University of São Paulo, São Paulo, Brazil
[6]Department of Biostatistics, Columbia University, New York, NY, USA
[7]Department of Ophthalmology, University of Montreal, Montreal, Que., Canada
[8]Department of Ophthalmology, Xin Hua Hospital affiliate of Shanghai Jiao Tong University School of Medicine, Shanghai, China.
[9]Department of Pathology & Cell Biology, Stem Cell Initiative (CSCI), Institute of Human Nutrition, College of Physicians and Surgeons, Columbia University, New York, NY, USA.
[10]Omics Laboratory, Byers Eye Institute, Department of Ophthalmology, Stanford University, Palo Alto, CA, USA
[*]Corresponding author

Running title: Cone Function in *USH2A* Patients

Address Correspondence:
Stephen H. Tsang, MD, PhD
Harkness Eye Institute
635 West 165th Street, Box 212, New York, NY 10032
Phone: (212) 342-1189 / Fax: 212-305-4987 / Email: sht2@columbia.edu

Supplemental Figure 1.

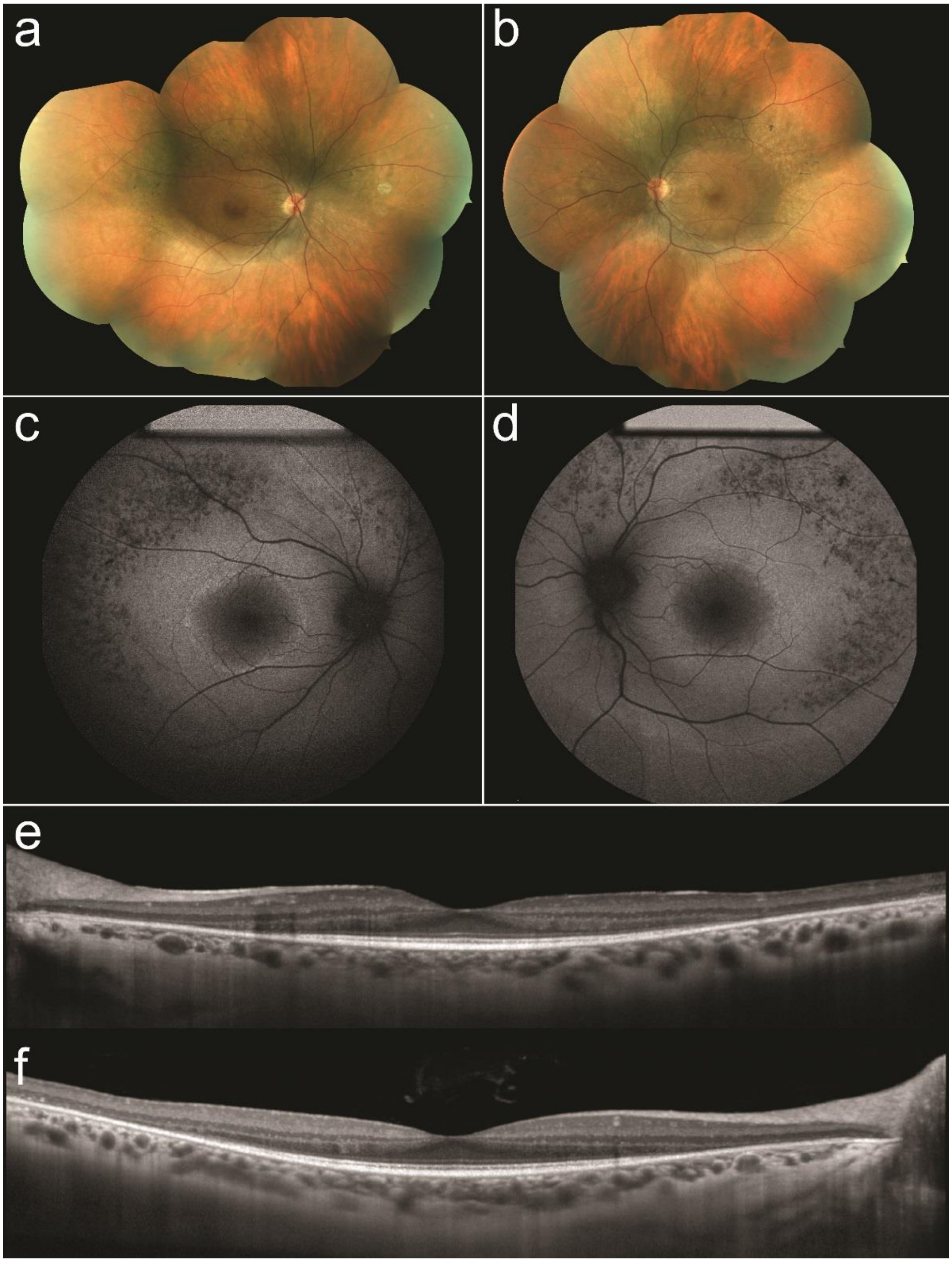

Supplemental Fig. 1. *Retinal imaging of an USH2A patient with normal audiometry.* Digital color fundus photography montage of the right (*a*) and left (*b*) eyes showing rare intraretinal pigment migration, pale discs, attenuated vasculature, and peripheral mottling. SW-AF imaging shows an autofluorescent ring in both eyes (*c*, *d*). SD-OCT of the right and left eyes (*e* and *f*) shows typical loss of outer nuclear layer in the periphery and sparing of the ellipsoid zone in the center macula.

Supplemental Table 1. All *USH2A* mutations in Patient Cohort

dbSNP	*Exon*	*Intron*	*Nucleotide change*	*Protein variant*	*Coding effect*
--	6		c.895delC	p.(Gln299Asnfs*37)	frameshift
rs397517963	6		c.1000C>T	p.(Arg334Trp)	missense
rs756570931	8		c.1478A>G	p.(Tyr493Cys)	missense
rs483353054	10		c.1724G>A	p.(Cys575Tyr)	missense
rs80338902	13		c.2276G<T	p.(Cys759Phe)	missense
rs80338903	13		c.2299delG	p.(Glu767Serfs*21)	frameshift
--	17		c3476C>T	p.(Pro1159Leu)	missense
--	17		c.3713C>G	p.(Thr1238Arg)	missense
--	19	19	c.4251+1G>A	p.(?)	--
--	22		c.4711G>C	p.(Ala1571Pro)	missense
--	29		c.5848A>G	p.(Thr1950Ala)	missense
--	30	30	c.6049+2T>G	p.(?)	--
--	42		c.8442_8443insT	p.(Thr2815Tyrfs*20)	frameshift
rs372347027	44	43	c.8682-9A>G	p.(?)	--
rs73090721	48		c.9459C>A	p.(Cys3153*)	nonsense
rs760858249	49		c.9676C>T	p.(Arg3226*)	nonsense
--	50	49	c.9740-1G>T	p.(?)	--
rs148660051	51		c.10073G>A	p.(Cys3358Tyr)	missense
rs202175091	54		c.10712C>T	p.(Thr3571Met)	missense
rs111033418	55		c.10759C>T	p.(Gln3587*)	nonsense
--	61		c.11918delC	p.(Ala3973Valfs*11)	frameshift
rs750396156	63		c.12574C>T	p.(Arg4192Cys)	missense
rs199605265	63		c.12575G>A	p.(Arg4192His)	missense
rs527236137	63		c.13010C>T	p.(Thr4337Met)	missense
rs768161313	63		c.13112_13115delAAAT	p.(Gln4371Argfs*19)	frameshift
--	63		c.13223T>C	p.(Val4408Ala)	missense
--	63		c.13231C>G	p.(Leu4411Val)	missense
--	63		c.13491_13499dupTACTCTCAC	p.(Thr4498_Thr4500dup)	in-frame
--	64		c.13943delG	p.(Gly4648Aspfs*30)	frameshift
rs397517990	65		c14287G>A	p.(Gly4763Arg)	missense
--	72	71	c.15520-22_15524del27	p.(?)	--

Supplemental Table 2. Functional and structural indices of a 41-year old female with normal audiometry and NSRP due to biallelic *USH2A* mutations.

**ffERG parameter**	**OD**	**OS**	**Comments**
Scotopic B-wave Amplitude (µv)	*122*	*143*	*dtl*
30 Hz Flicker Amplitudes (µv)	*52*	*74*	*dtl*
30 Hz Flicker Implicit Time Delay (ms)	*31*[d]	*32*[d]	*dtl*
Ellipsoid Zone-line Length (µm)	*3661*	*3810*	--
Horizontal ring diameter (µm)	*4784*	*4564*	--
Vertical Ring Diameter (µm)	*4145*	*3822*	--
